# Derivative Texture Analysis of Tumor Histology for Evaluating Ultrasound Nanobubble-Driven Radiation Enhancement

**DOI:** 10.3390/cells15161491

**Published:** 2026-08-19

**Authors:** Yingqi Zhang, Lakshmanan Sannachi, Kai Xuan Leong, Wenyi Yang, Deepa Sharma, Ryan Yang, Gregory J. Czarnota

**Affiliations:** 1 Physical Sciences, Sunnybrook Research Institute, Toronto, ON M4N 3M5, Canada; 2 Department of Radiation Oncology, Sunnybrook Health Sciences Centre, Toronto, ON M4N 3M5, Canada; 3 Department of Radiation Oncology, University of Toronto, Toronto, ON M5S 1A1, Canada; 4 Department of Medical Biophysics, University of Toronto, Toronto, ON M5S 1A1, Canada

**Keywords:** radiation, nanobubble, ultrasound, derivative texture analysis, tumor

## Abstract

Microbubbles (MBs) have been used as a radiosensitizer, and their combination with ultrasound (US) has emerged as a promising strategy to improve radiotherapy outcomes in tumor treatment. Nanobubbles (NBs), which are about 1000 times smaller than MB and have enhanced stability, have been considered to have the potential for further radiosensitization enhancement. In this study, tumor-bearing models were treated with and without nanobubble–ultrasound (NBUS) treatment before radiotherapy (XRT) to evaluate the radiosensitization. The time intervals between the NB injection and US exposure, and between US and XRT, were optimized to maximize the therapeutic efficacy. A derivative texture analysis was applied to extract microstructural features from haematoxylin and eosin (H&E)-stained images. A one-way ANOVA test combined with a k-NN classifier and Tukey’s Honestly Significant Difference (HSD) test was used to identify the best features for assessing treatment outcomes. These were applied to H&E-stained tumor sections for evaluating microstructural alterations associated with NB-driven radiosensitization. The results indicated that both the 2 Gy and 8 Gy radiation groups showed enhanced treatment outcomes when NBUS therapy was administered before radiotherapy. Notably, NBUS + 2 Gy could achieve outcomes comparable to 8 Gy only. Additionally, a derivative texture analysis was demonstrated to be a promising and powerful technique for analyzing H&E images. This study provides a quantitative framework for assessing nanobubble-enhanced radiotherapy.

## 1. Introduction

Radiation therapy has been widely used in clinical use and it is still one of the most effective modalities for cancer treatment [1]. Around 50–60% of patients with cancer are receiving radiation treatment during their cancer treatment process [2]. Radiation treatment primarily impairs tumor cell proliferation by breaking DNA double strands and radiation can cause apoptosis by generating free radicals that lead to double-stranded DNA breaks [3,4]. The accumulation of this damage prevents cancer cells from replicating, leading to cell death and inhibiting the tumor’s further growth [5,6,7]. However, the outcomes of the radiation treatment in clinical practice are sometimes limited by radioresistance, especially in hypoxic tumor regions where the deficiency of oxygen can reduce the fixation of radiation-induced DNA damage [7,8]. Several strategies have been investigated to address radioresistance, such as combining radiotherapy with radiosensitizing agents that could inhibit DNA repair or block survival pathways [9,10]. In addition, some advanced radiation modalities, such as proton and heavy-ion therapy, and adaptive radiotherapy, such as modifying doses or treatment plans based on the tumor response, have demonstrated promising potential to overcome radioresistance [11,12]. However, strategies to enhance the sensitivity of radiation treatment while reducing the damage to the normal tissue are still one of the biggest challenges of translational research.

Microbubbles (MBs) are gas-filled vesicles stabilized by a shell which is composed of lipids, polymers, or proteins. MBs have been used as a ultrasound-responsive agent in various biomedical applications for many years [13,14,15]. MBs’ diameter is typically 1–10 μm and is considered as one of the FDA-approved ultrasound contrast agents due to its strong echogenicity [13,16]. However, MBs still face several challenges in clinical use. Their relatively large size and short circulation half-life, leading to rapid clearance by the liver and spleen, limit their uses in extravascular imaging and therapeutic delivery [17,18]. In order to overcome these limitations, researchers have recently explored the use of nanobubbles (NBs) [19]. NBs are nanoscale, gas-filled cavities typically encapsulated by a shell composed of lipids, polymers, or surfactants [19,20]. They are generally defined as having diameters in the submicron range, mostly between 150–500 nm, which are small enough to navigate biological barriers and respond to ultrasound stimulation [19,21]. Compared with MBs, their smaller size provides enhanced stability and a longer circulation time [22]. In tumor treatment, NBs can more effectively accumulate within the tumor vasculature through the enhanced permeability and retention (EPR) effect, enabling deeper penetration into tissue [23,24]. Currently, NBs are being explored in various tumor therapy applications; the combination of using MBs/NBs with ultrasound has been approved to enable therapeutic interactions with surrounding tissues [25,26]. When MBs/NBs are activated by ultrasound, the oscillation of bubbles and the collapse of the encapsulated gas may lead to acoustic cavitation. Stable cavitation can produce periodic oscillations and the inertial cavitation can induce violent collapse with generating microjets, localized shock waves, and shear stress [27]. Basically, NBs have the great potential to directly interact with tumor cells, rather than only acting on blood vessels like MBs, which may enhance the radiosensitivity of tumor cells more than MBs.

Texture analysis is an image analysis method used for quantifying patterns, microstructures, and spatial variations in images [28,29]. Currently, texture analysis techniques are widely used in material science, remote sensing, computer vision, and medical imaging [30,31]. Texture analysis is not only just focused on analyzing pixel intensities directly but also on how those intensities are arranged (such as measuring contrast, coarseness, directionality, regularity, and granularity), allowing it to detect subtle differences in patterns when they are imperceptible [28,32]. The Gray-Level Co-occurrence Matrix (GLCM), Gray Level Dependence Matrix (GLDM), Gray Level Size Zone Matrix (GLSZM), and Neighbouring Gray Tone Difference Matrix (NGTDM) are involved in the analysis [33,34]. Different methods provide different purposes for the analysis. As compared in Table 1, the GLCM is usually used to detect fine texture differences or edge patterns, the GLDM is usually used to measure the texture granularity or coarseness, the GLSZM is usually used to analyze the structure size and uniform regions, and the NGTDM is usually used to measure the overall texture contrast and smoothness [34,35,36,37,38]. In summary, the GLCM and the GLDM are good at capturing fine-grained and localized features, where the GLSZM and the NGTDM extend the analysis to the regional and global scales [39]. However, each method alone provides only a partial perspective, and their combined use, derivative texture analysis, offers great potential for achieving a more comprehensive and reliable characterization of the texture.

Derivative texture analysis is a higher-order texture analysis technique, where the second-order texture analysis is applied to the previous derived texture map rather than the original image. The core idea is to treat the first-order texture feature as a new image and then extract higher-order texture features from it. Derivative texture analysis is an advanced approach that focuses on how texture features change spatially, rather than just measuring their absolute values simply [40,41,42]. Moreover, this approach is analogous to the multi-level or pyramid feature extraction in deep learning, as it enhances the sensitivity to local and higher-order changes that may remain undetected through a single round of texture characterization [42]. H&E staining is a part of routine pathological assessment, so derivative texture analysis could be incorporated into pathology workflow without additional staining or specific molecular assays. Meanwhile, it may work as an objective and quantitative tool for optimizing treatment and serve as candidate biomarkers for early response assessment. Furthermore, these features could be used for supporting the future translation of personalized treatment planning and monitoring the response in clinical practices.

Bubble effects on tissue are complex especially when combined with radiotherapy as different tissue areas are imparted different types of damage. Texture-based image analysis has been successfully applied to evaluate the features of histopathology in response to MB-enhanced radiotherapy [43]. In the work here, we specifically develop texture-methods to evaluate the effects of NBs combined with radiotherapy on histopathological features in animals bearing tumors.

## 2. Materials and Methods

### 2.1. Animal Model and Experimental Design

All animal experiments were conducted following national guidelines set by the Canadian Council on Animal Care and upheld by the Sunnybrook Research Institute Animal Care Committee. RPMI-1640 (ATCC, Manassas, VA, USA) media supplemented with 10% characterized fetal bovine serum (Sigma Aldrich, St. Louis, MO, USA) and 1% penicillin/streptomycin antibiotic (Sigma Aldrich, St. Louis, MO, USA) were used to culture the Human PC3 prostate cancer cells (American Type Culture Collection, Manassas, VA, USA). Cells were prepared in Dulbecco’s Phosphate-Buffered Saline (DPBS) for the injection. Then, 100 μL 1 × 10^6^ cells/mL cells were injected into the hind leg of SCID mice (Charles River Inc., Wilmington, MA, USA). Tumors were permitted to reach 8–10 mm over a period of 3 weeks before treatment.

**Figure 1:**
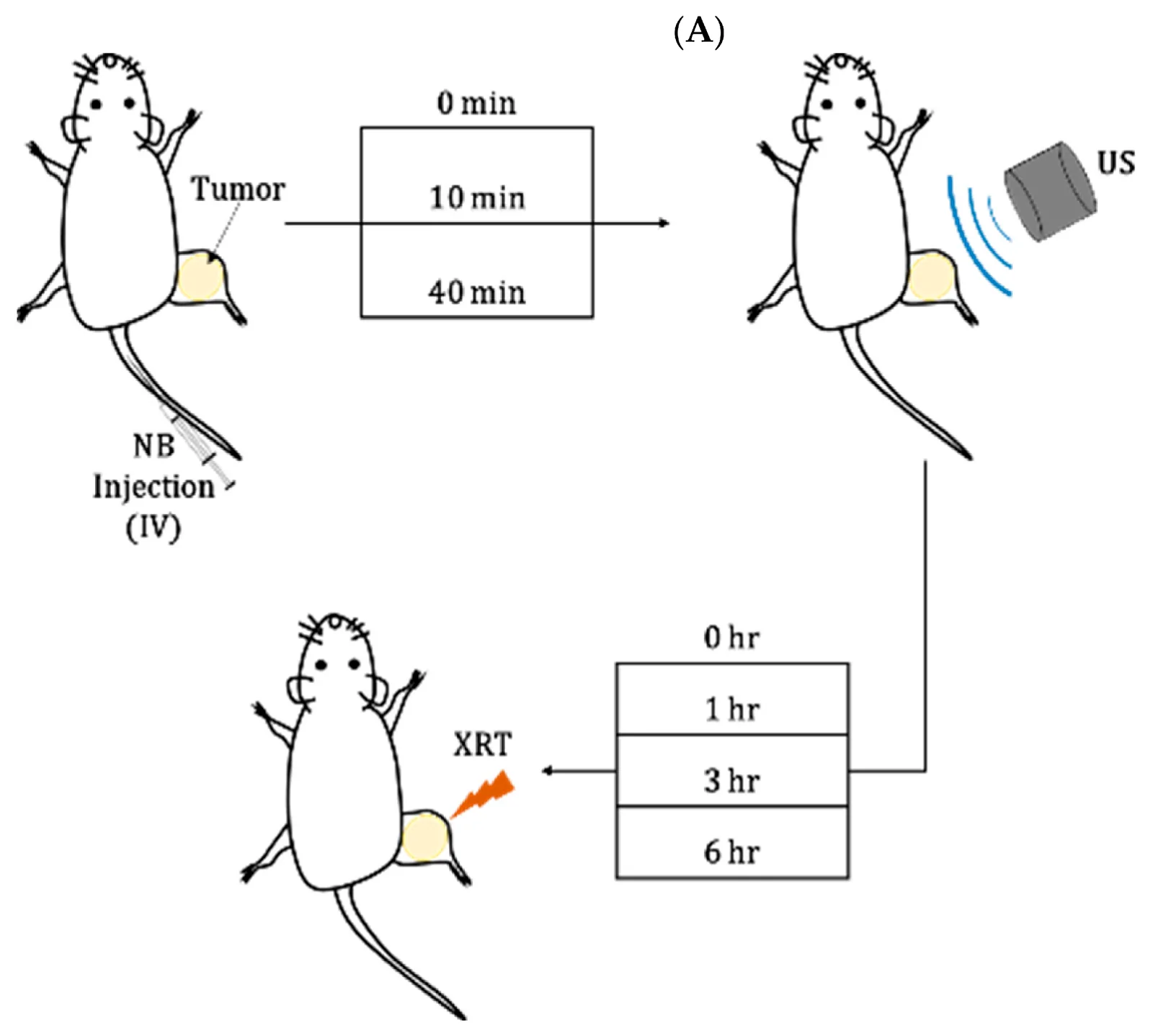

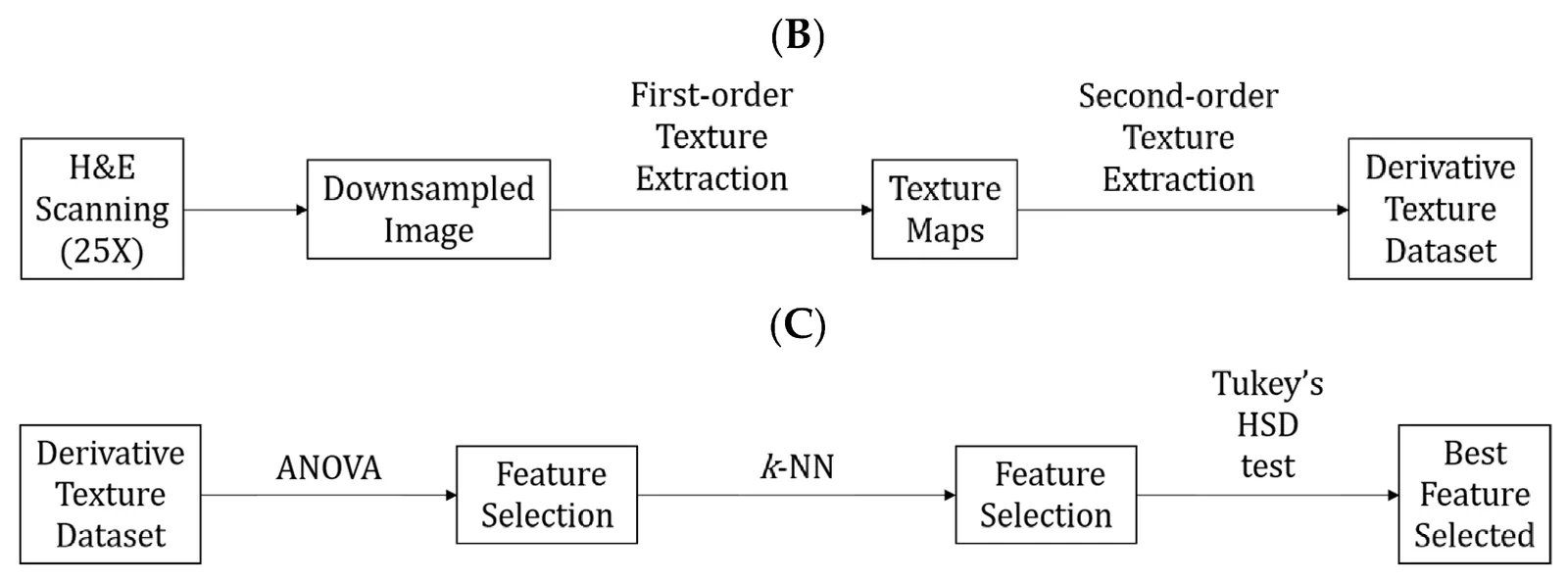
(**A**) Schematic of experimental process. (**B**) Scheme of derivative-texture-based image analysis. (**C**) Scheme of feature selection approach.

Mice bearing prostate cancer (PC3-cell line) xenografts were assigned to one of the following treatment groups: control, radiation only (2 Gy/8 Gy), NBs with ultrasound (NB + US) only, and combined treatment (NB + US + 2 Gy/8 Gy). As illustrated in Figure 1A, tumor-bearing mice were anesthetized with 2% isoflurane prior to treatment. A 100 µL bolus of NBs was injected using a tail-vein catheter, followed by a 150 µL saline flush. NB formulation and production is detailed in Hysi’s work [44]. Mice were positioned with their lower body submerged in a 37 °C water bath, and the tail was elevated to ensure proper acoustic coupling. The therapeutic ultrasound transducer (500 kHz single-element transducer IL0509HP, Valpey-Fisher, MA, USA) was set aligned with the tumor-bearing limb at a focal distance of 8.5 cm. NBs were activated under ultrasound for 5 min with a peak negative pressure of 570 kPa. Radiation therapy was subsequently administered using a Faxitron cabinet device (Faxitron X-Ray LLC, AZ, USA) operating at 160 kVp with animals positioned 30 cm away from the X-ray source. The time interval (0 min, 10 min, and 40 min) between NB injection and ultrasound treatment, and the time interval (0 h, 1 h, 3 h, and 6 h) between ultrasound treatment and radiation exposure were tested in this study, and, based on the results, the optimal intervals were determined in order to achieve maximal therapeutic enhancement in future use of NBUS to achieve maximal therapeutic enhancement. Three activation timepoints (0 min, 10 min, and 40 min) were used to evaluate the expected progression of vascular response, nanobubble distribution, and tumor response after treatment. Early activation (0 min) was selected to primarily reflect immediate vascular effects, while later activation (10 min and 40 min) was selected to evaluate the impact of progressive distribution and potential tumor cell targeting. Meanwhile, the interval between NBUS treatment and radiation delivery (0, 1, 3, and 6 h after NBUS treatment) was investigated to evaluate how the NBUS treatment and subsequent radiation therapy can affect the enhanced-treatment-induced cell status changes. This design was intended to provide mechanistic insight into how interval between administration and activation contributes to treatment efficacy. A total of 24 experimental groups were evaluated, involving 92 animals in total. The detailed treatment combinations and animal distributions are summarized in Table 2. Animals were euthanized 24 h after treatment, and the tumors were excised. Tumors were stored in formalin (10%) overnight before further processing. Tumors were fixed, paraffin-embedded, sectioned at 5 µm thickness, and stained with H&E.

### 2.2. Derivative Texture Feature Extraction and Selection

The derivative texture feature extraction approach is presented in Figure 1B. H&E-stained slides were digitally scanned at optical 25× magnification (with a pixel size of 0.4 µm × 0.4 µm), and, then, first, downsampled 10 times to generate lower-resolution images for subsequent texture feature extraction (with a pixel size of 4 µm × 4 µm). For the texture analysis, the entire tumor cross-section was manually contoured as a single region of interest (ROI) using a custom MATLAB/Python-based workflow, the necrotic regions were included in the ROI for remaining overall histopathological structure of the tumor. The analysis was based on entire tumor selection, so the subjective selection of preferred regions can be avoided. From the downsampled images, the first-order texture features were extracted and used to generate initial texture maps using four radiomic methods: Gray Level Co-occurrence Matrix (GLCM), Gray Level Dependence Matrix (GLDM), Gray Level Size Zone Matrix (GLSZM), and Neighboring Gray Tone Difference Matrix (NGTDM). The second-order texture features were then derived from these processed texture maps using a second-pass texture analysis to capture more complex relationships between pixel intensities. Here, the feature selection process is illustrated in Figure 1C. A subset of derivative features was selected from 1053 derivative feature candidates based on one-way ANOVA test (*p* < 0.05). Then, the optimal features were identified based on the results of classification accuracy using k-NN classifier model. Finally, the best feature was selected by Tukey’s Honestly Significant Difference (HSD) test. For tumor ROI segmentation and quantitative texture analysis, the custom software was developed using MATLAB (version 2020b; MathWorks, Natick, MA, USA) and Python (version 3.13.5; Python Software Foundation). Specifically, texture features were determined from the original H&E-stained images using four quantitative methods: GLCM (24 features), GLDM (14 features), GLSZM (16 features), and NGTDM (5 features). After completing the first-order texture analysis, the resulting texture maps underwent second-order texture analysis to derive the corresponding derivative texture features. In total, 1053 derivative texture features were obtained. An overall one-way analysis of variance (ANOVA) among different groups (Control–NBUS–2Gy–NBUS2Gy) was applied to identify features with significant differences among treatment groups. In addition, following the one-way ANOVA test for feature selection, a *k*-NN classifier model was applied to identify the most informative features or combinations of features, to distinguish between treatments across different samples. Here, the parameter K was tested from 1 to 4, which specifies the number of nearest neighbors considered by the algorithm when making a prediction. In addition, the number of feature combinations was tested from 1 to 8 in order to evaluate the results. Specific attention was focused on Inverse Variance, a texture feature that was used that measures local homogeneity of gray-level intensities. Image regions with smooth textures (similar pixel intensity) usually show higher values, while regions with heterogeneous texture (high intensity variability) usually show lower values. Large Area Emphasis (LAE) quantifies the proportion of large homogeneous zones in the image. Well-differentiated tissue with less heterogeneity typically produces higher values, whereas fragmented and heterogeneous tissue typically results in lower values. Large Area High Gray Level Emphasis evaluates the large homogeneous zones with high intensity. Viable and dense tumor tissue, reflecting less necrosis or cell death, results in higher values, while fragmented, heterogeneous, or necrotic tissue, which is more consistent with increased tumor cell death, results in lower values. The selected feature was used as a quantitative biomarker to evaluate the treatment efficacy. Since it can provide a sensitive measure of treatment-induced cell status changes, we evaluated the treatment outcomes and identifies difference in the treatment response by comparing feature values across different treatment groups.

## 3. Results

Texture and texture-derivative features were calculated in a number of different manners. As shown in Table 3, 32 features were identified as significant between groups (*p* < 0.05). Among these, the feature 20 (glszm_LargeAreaEmphasis-glszm_SmallAreaEmphasis) showed the lowest *p*-value (*p* = 1.18 × 10^−6^). Furthermore, all treatment groups differed significantly from the control group, with *p*-values of 3.15 × 10^−4^, 1.08 × 10^−3^, and 4.57 × 10^−7^ for control versus NBUS, control versus 2 Gy, and control versus NBUS + 2 Gy, respectively. In addition, significant differences were observed between NBUS + 2 Gy and NBUS, and between NBUS + 2 Gy and 2 Gy, with *p*-values of 1.70 × 10^−2^ and 4.61 × 10^−3^, respectively, indicating that the combination treatment (NB + US + XRT) produced a significant difference in therapeutic effect. For analysis purposes, we selected the one best feature which discriminated between all groups in order to identify a characteristic that best tracked the differences between all groups and was concordant with potential cell destruction as assessed using the standard histological interpretation.

A more extensive search of features that were distinct and linked to the response was also carried out using the KNN methodology for multi-variate analysis. For the KNN analysis of the most differentiating features, the predicted treatment outcomes are summarized in Table 4 which illustrates the effect of using different numbers of features and different K values in the *k*-Nearest Neighbors (*k*-NN) algorithm to assess the classification accuracy. Table 4A shows the effect of using one to eight features on evaluating the accuracy when K = 1. The highest accuracy, 80.9%, occurs with using a single feature (feature 20). For K = 1, more features appeared to introduce noise or irrelevant information, reducing the performance of the model, with the accuracy dropping to 42.9% when using a combination of eight features. Table 4B examines the accuracy for one to eight features with K = 2. This also used a combination of four features (feature 1, feature 19, feature 20, and feature 21), and obtained an equivalent top accuracy of 80.9%, which was the same as using feature 20 alone. However, the accuracy dropped most when combined using seven to eight features. For K = 2, a small subset of features worked best. More feature combinations reduce the performance. For K = 3, the accuracy fluctuates, with the number of features in the combination increasing as shown in Table 4C. However, feature 20 consistently demonstrated its importance. For K = 4, a small and selected set of feature combinations works better than the single-feature or the all-features combination. As shown in Table 4D, the combination of three features (feature 20, feature 21, and feature 31) and the combination of four features (feature 1, feature 19, feature 30, and feature 32) both reached an accuracy of only 71.4%.

For the multivariate data, the sensitivity (Sn), specificity (Sp), and precision were computed within each group. Meanwhile, the “macro” average (the average of metrics across all classes) and the “micro” average (which aggregates the false positive (FP), true positive (TP), false negative (FN), and true negative (TN) across classes, and then calculates the metrics) across all groups were also obtained and are presented in Table 5. Table 5A displays the performance of applying the *k*-NN classifier model to the dataset based on using the best single feature (feature 20), and Table 5B presents the performance of applying the *k*-NN classifier model to the dataset based on a combination of four selected features.

**Figure 2:**
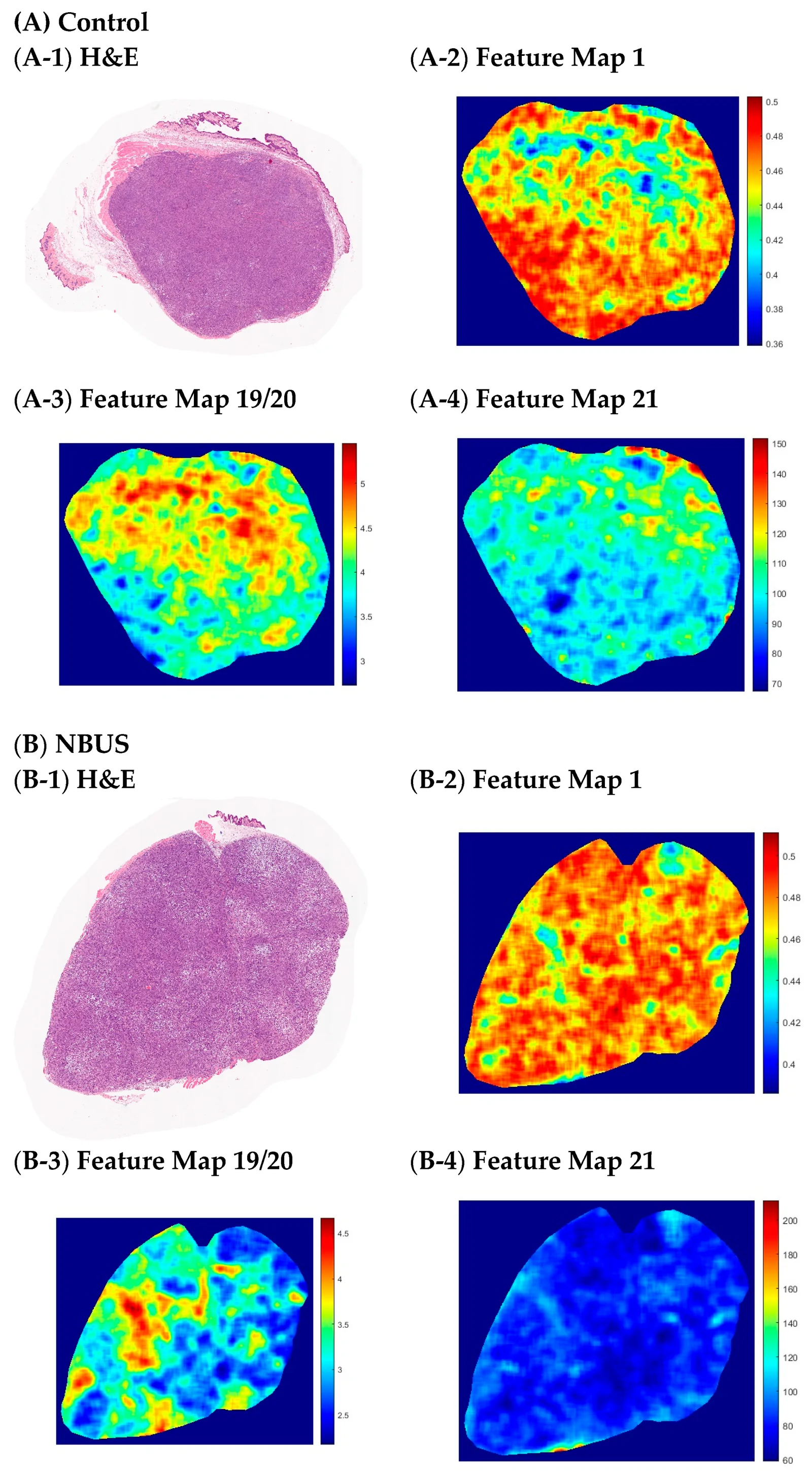

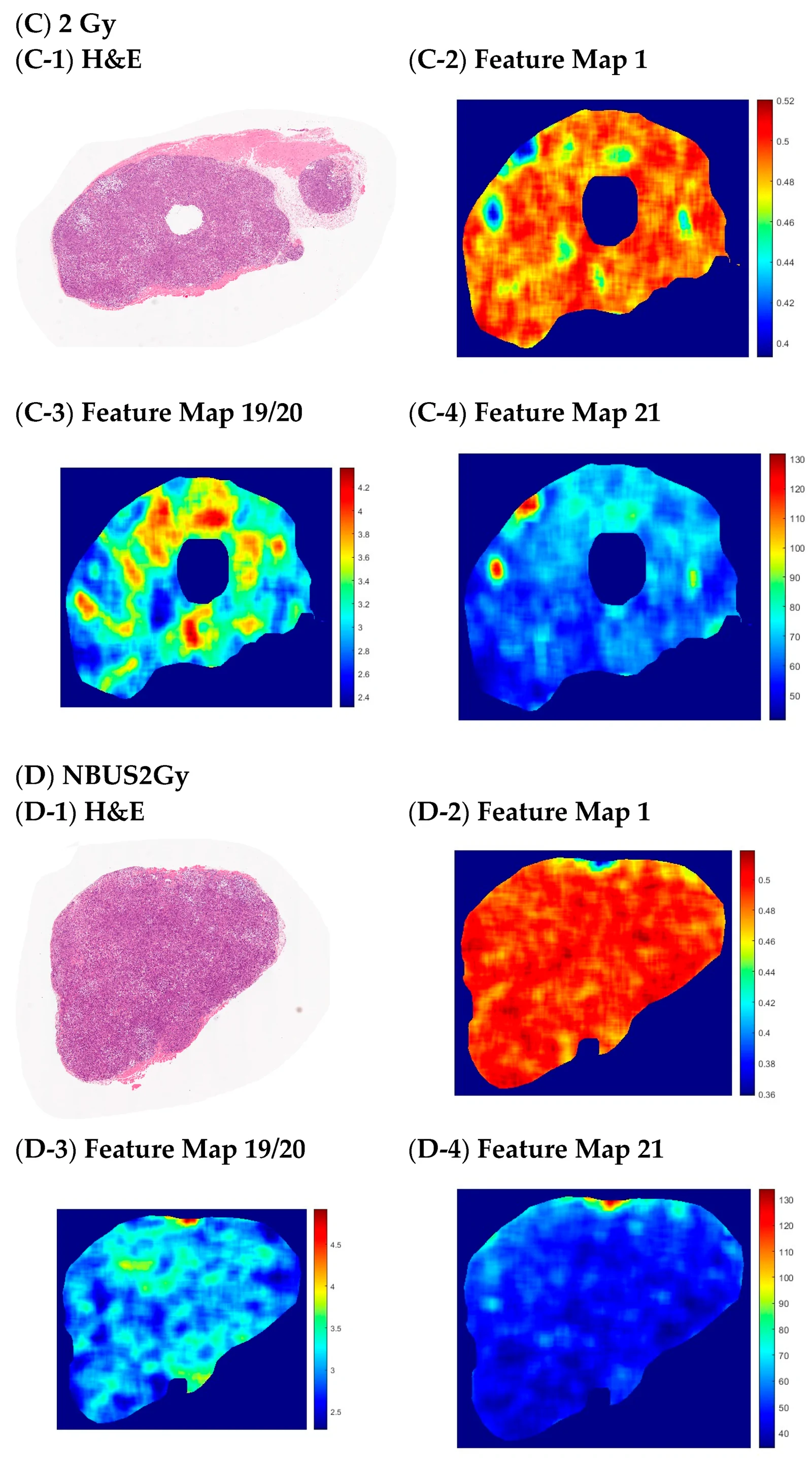
Comparison of H&E and selected feature mapping: (**A**) control: (**A-1**) H&E, (**A-2**) feature map 1, (**A-3**) feature map 19/20, (**A-4**) feature map 21; (**B**) NBUS: (**B-1**) H&E, (**B-2**) feature map 1, (**B-3**) feature map 19/20, (**B-4**) feature map 21; (**C**) 2 Gy: (**C-1**) H&E, (**C-2**) feature map 1, (**C-3**) feature map 19/20, (**C-4**) feature map 21; (**D**) NBUS2Gy: (**D-1**) H&E, (**D-2**) feature map 1, (**D-3**) feature map 19/20, and (**D-4**) feature map 21.

Figure 2 presents a comparison between H&E-stained tissue sections and selected feature maps generated from first-order texture feature determination (glcm_InverseVariance (feature 1), glszm_LargeAreaEmphasis (feature 19 and feature 20), and glszm_LargeAreaHighGrayLevelEmphasis (feature 21)) across the four experimental groups (Control, NBUS, 2 Gy, and NBUS + 2 Gy). The control group showed preserved tissue morphology without evident pathological alterations. Both the NBUS-treated group and the 2 Gy-treated group maintained an overall intact structure but showed obvious structural disorganization compared with the control group. In contrast, the NBUS + 2 Gy group exhibited the most pronounced histological changes, with a visually obvious disrupted architecture and denser staining compared to the other groups.

Three representative feature maps (feature 1, feature 19 and feature 20, and feature 21) were analyzed across all groups to evaluate the spatial heterogeneity and intensity distribution. Based on the results shown in Figure 2, although the map of feature 1-glcm_InverseVariance varied among different groups, it did not respond well to the cell status. Both the feature 19/20-glszm_LargeAreaEmphasis map and feature 21-glszm_LargeAreaHighGrayLevelEmphasis maps corresponded to regions of cell death. However, the map of feature 21 was less sensitive than the map of feature 19/20, as it cannot distinguish the differences between the NBUS group and the NBUS + 2 Gy group. Thus, the map generated by feature 19/20, glszm_LargeAreaEmphasis, was used for further derivative analysis, with the red and blue regions corresponding to viable and dead tumor cells, respectively. In addition, the yellow and green regions correspond to apoptotic areas.

**Figure 3:**
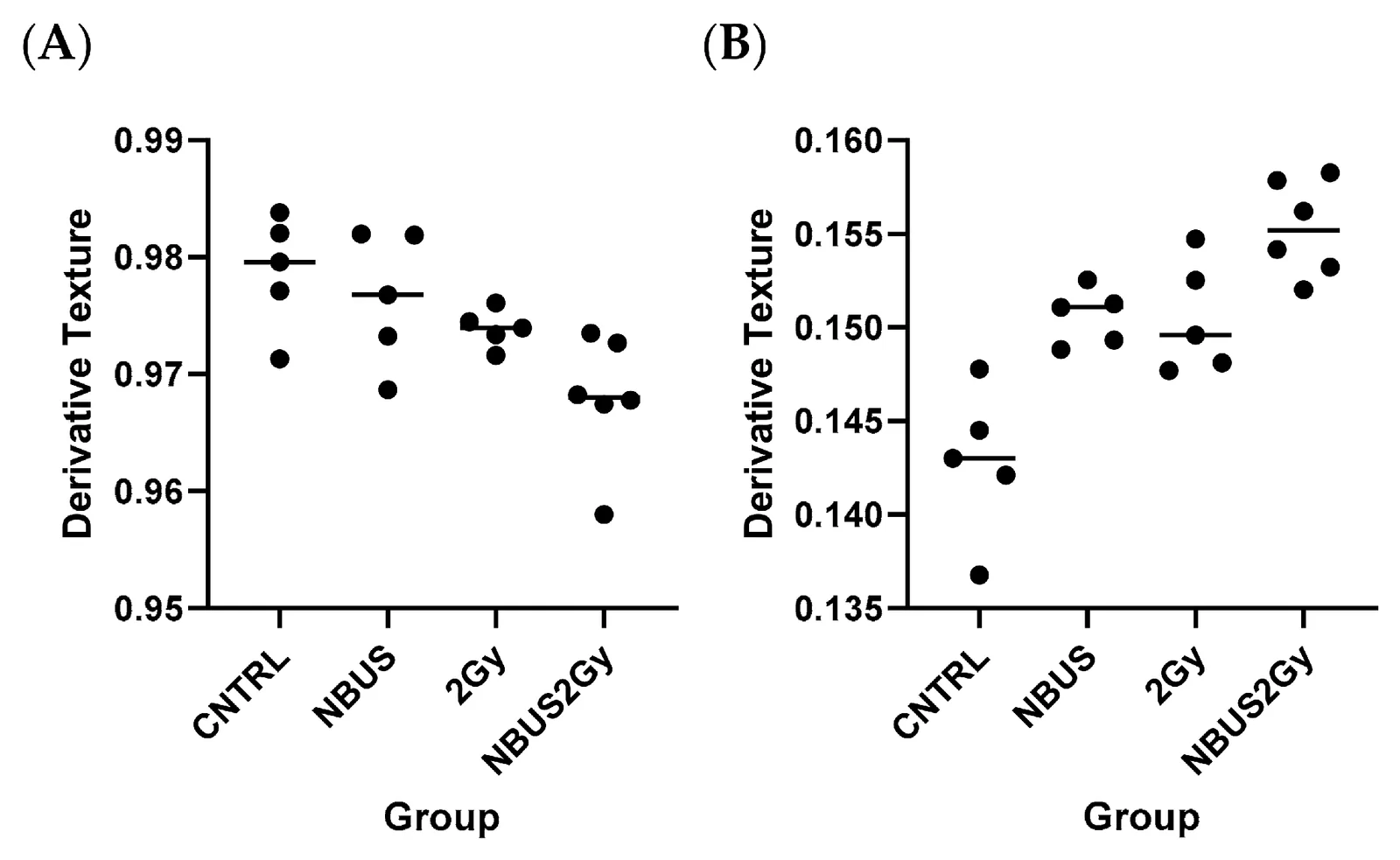

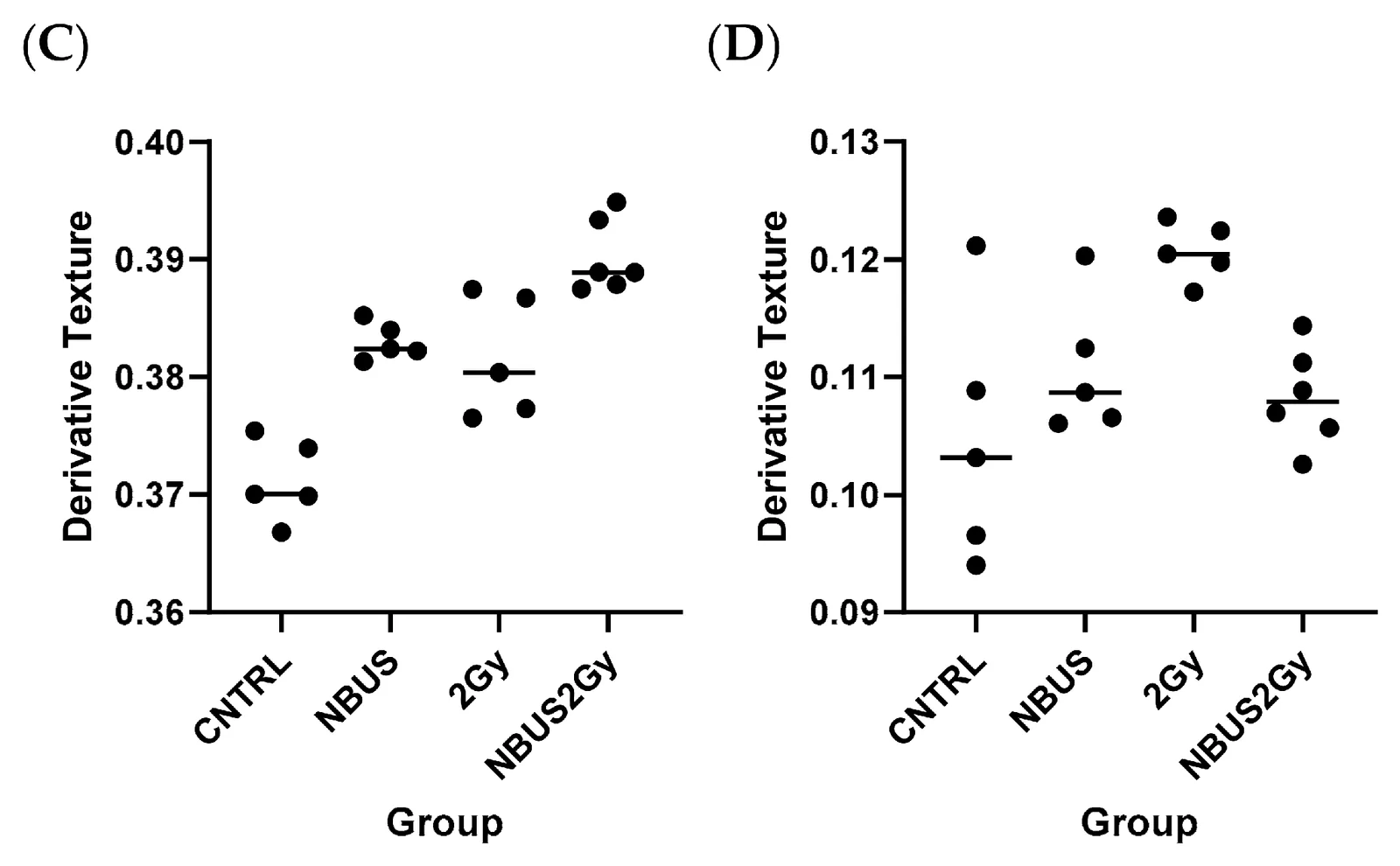
Plotting of selected radiomic features. (**A**) glcm_InverseVariance-glcm_Correlation; (**B**) glszm_LargeAreaEmphasis-glszm_SizeZoneNonUniformityNormalized; (**C**) glszm_LargeAreaEmphasis-glszm_SmallAreaEmphasis; and (**D**) glszm_LargeAreaHighGrayLevelEmphasis-glszm_SizeZoneNonUniformityNormalized.

An analysis of the selected radiomic features revealed significant differences among the experimental groups (Figure 3). As shown in Table 6, the feature 19 (glszm_LargeAreaEmphasis-glszm_SizeZoneNonUniformityNormalized) and the feature 20 (glszm_LargeAreaEmphasis-glszm_SmallAreaEmphasis) presented strong group-dependent variation, with a dramatic increase in the NBUS group and the 2Gy group and significantly higher values in the NBUS + 2Gy group compared to controls (*p* < 0.01). Tukey’s multiple-comparisons test also indicated significant differences between the NBUS group and the 2 Gy group and the NBUS + 2 Gy group. However, the feature 1 (glcm_InverseVariance-glcm_Correlation) demonstrated a similar trend in the analysis as feature 19 and feature 20, but an excessive overlap resulted in a lower sensitivity, and the feature 21 (glszm_LargeAreaHighGrayLevelEmphasis-glszm_SizeZoneNonUniformityNormalized) failed to distinguish the differences between each group, making both of them unsuitable for practical uses. These findings suggest that specific radiomic texture features are promising to group differences and the feature 20 (glszm_LargeAreaEmphasis-glszm_SmallAreaEmphasis), with the highest sensitvity, may serve as the most sensitive imaging biomarker for the treatment response.

The Large Area Emphasis (LAE), derived from the GLSZM, was used to characterize the spatial homogeneity and structural continuity of H&E-stained tumor tissue. Higher LAE values usually correspond to large, spatially coherent regions with uniform intensity distributions, which reveal a more homogeneous and structurally organized tissue structure. In contrast, lower LAE values indicate fragmented, heterogeneous regions with a disrupted structural continuity and increased textural complexity.

**Figure 4:**
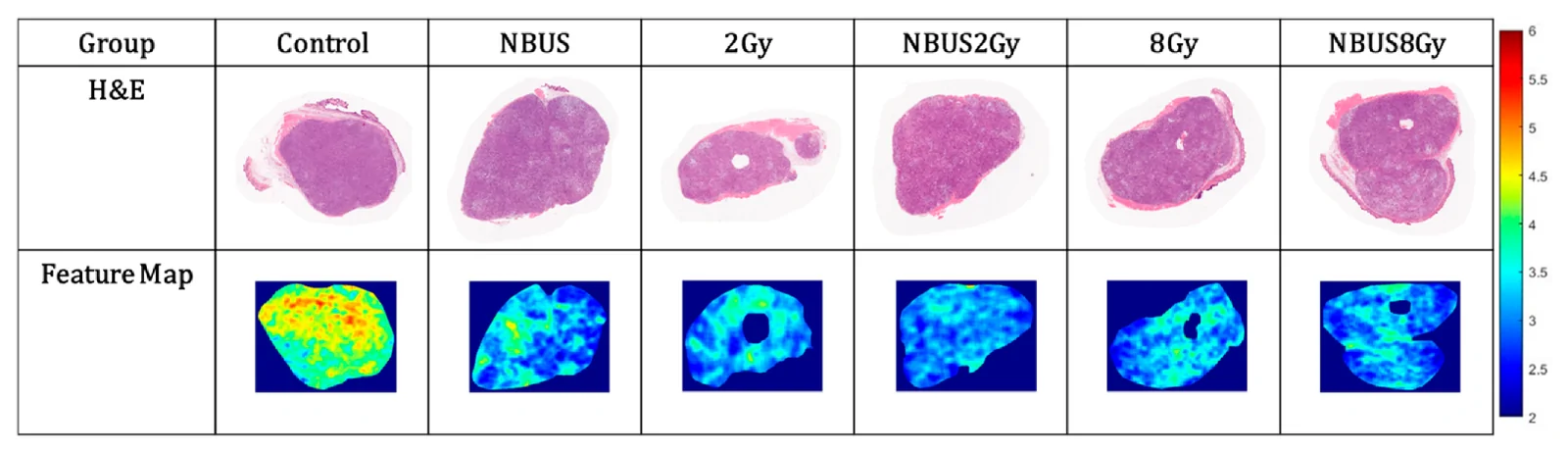
Histological and feature map of tumor tissues under different treatment conditions (normalized).

Based on this feature, a comparison of the GLSZM_LAE texture feature maps generated from H&E-stained tumor images with their corresponding histological sections was carried out. This indicated that treatment effects could be visually inferred from the visualized patterns in the feature maps. Regions of the tumor color-coded (red corresponding to higher GLSZM_LAE values) indicated areas with a higher structural uniformity, whereas regions appearing with lower GLSZM_LAE values (blue) indicated areas with a greater cellular heterogeneity. Meanwhile, biologically, a higher GLSZM_LAE reflected the presence of large and uniform regions of similar intensity, consistent with a more observed homogeneous tissue organization, whereas a lower GLSZM_LAE value corresponded to an increased microstructural heterogeneity, consistent with observations of cellular disruption, necrosis, or apoptosis. Specifically, blue regions (lower LAE) correspond to necrotic areas, characterized in H&E-stained images which the loss of cellular architecture, nuclear breakdown, or disappearance. Yellow to green regions represent an intermediate state, such as apoptotic or hypoxic stress regions, which are associated with inflammatory cell infiltration, a reduced cell density, and a partial loss of structural integrity. These regions usually show irregular cell shapes, nuclear condensation or fragmentation, and the loss of tissue architectures. Red regions (higher LAE) correspond to viable tumor cells, characterized by a higher cellular density, hyperchromatic enlarged nuclei, and a relatively preserved and continuous tissue architecture. Figure 4 presents representative H&E-stained tumor sections (top row) and their corresponding computational feature maps (bottom row) under different experimental conditions, including Control, NBUS, 2 Gy, NBUS + 2 Gy, 8Gy, and NBUS + 8 Gy. The H&E-stained sections illustrate distinct morphological variations across treatment groups, while the GLSZM_LAE-derived feature maps highlight the quantitative differences in tissue heterogeneity and treatment-induced microstructural alterations.

In H&E-stained histology images, the control group showed a preserved tissue architecture with dense cellularity, which represents an untreated tumor morphology. The NBUS group demonstrated modest disorganization compared to the control, indicating mild structural effects induced by NBUS only. Following irradiation (2 Gy and 8 Gy), the dose-dependent histopathological changes were observed, including a reduced cellular density and disrupted tissue structure, more prominent in the 8 Gy group. In the tumor texture maps, the encoded red and yellow regions, representing areas of higher GLSZM_LAE values and greater tissue homogeneity, decreased after treatment, indicating structural disruption and increased heterogeneity within the tumor tissue. Comparing 2 Gy and NBUS + 2 Gy, both histological and computational analyses demonstrated an enhanced radiotherapy response when NB were used, as evidenced by the marked reduction in red and yellow regions (indicating lower GLSZM_LAE values and increased tissue heterogeneity) in the feature maps. Similarly, a comparison between 8G y and NBUS + 8Gy demonstrated a similar NBUS enhanced response to radiation.

The combination treatments (NBUS + 2 Gy and NBUS + 8 Gy) demonstrate a clear synergistic interaction between NBUS and radiotherapy. However, compared with the group control (0.37122 ± 0.00345), based on the relative enhancement ratios between 2 Gy (0.38024 ± 0.00463) and NBUS + 2 Gy (0.39070 ± 0.00313) and between 8 Gy (0.38758 ± 0.00263) and NBUS + 8 Gy (0.38714 ± 0.00647), there appears to be a limit to the magnitude of this enhancement, suggesting that NBUS-induced radiosensitization may be limited beyond a certain radiation dose threshold.

**Figure 5:**
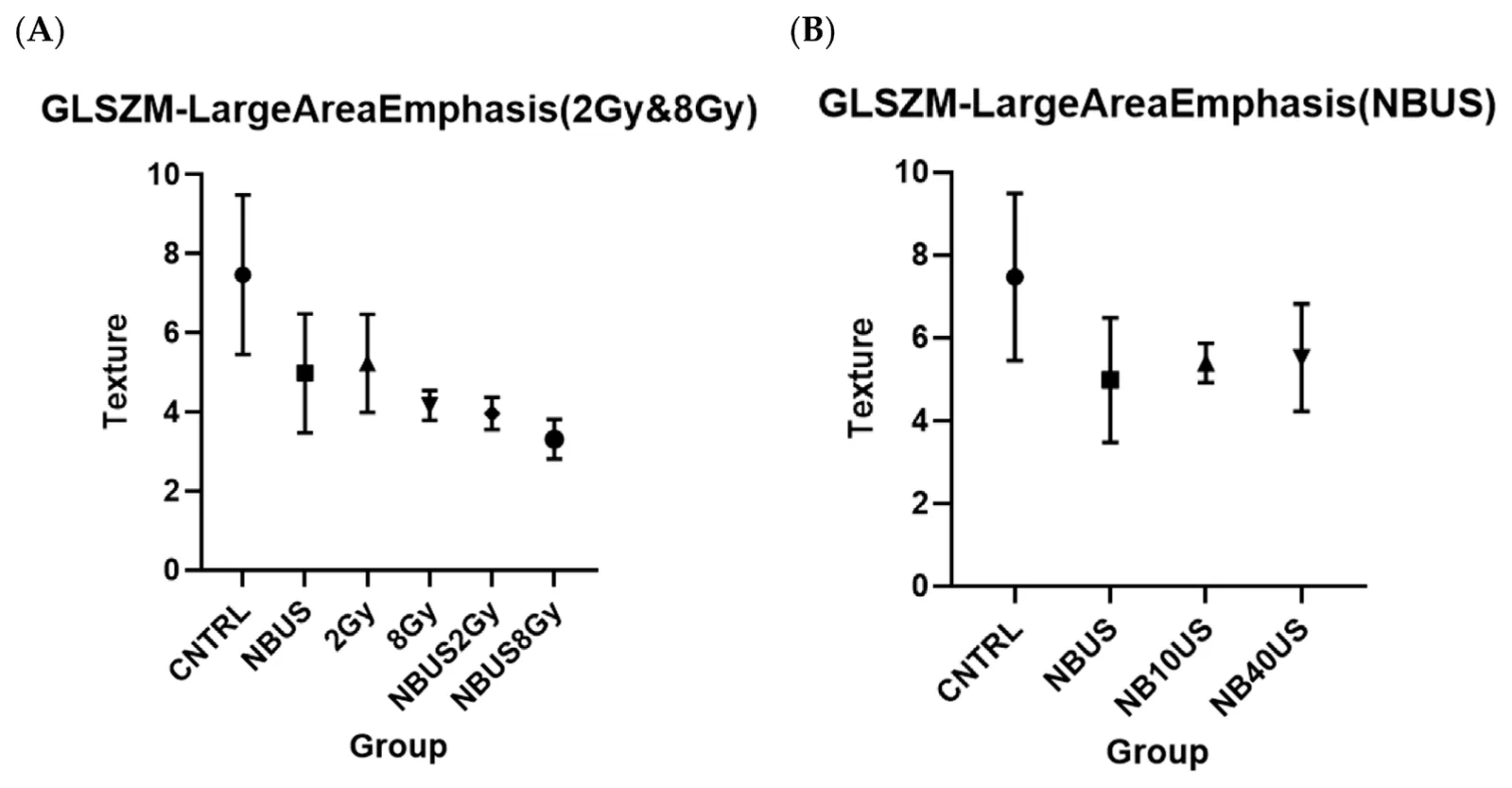

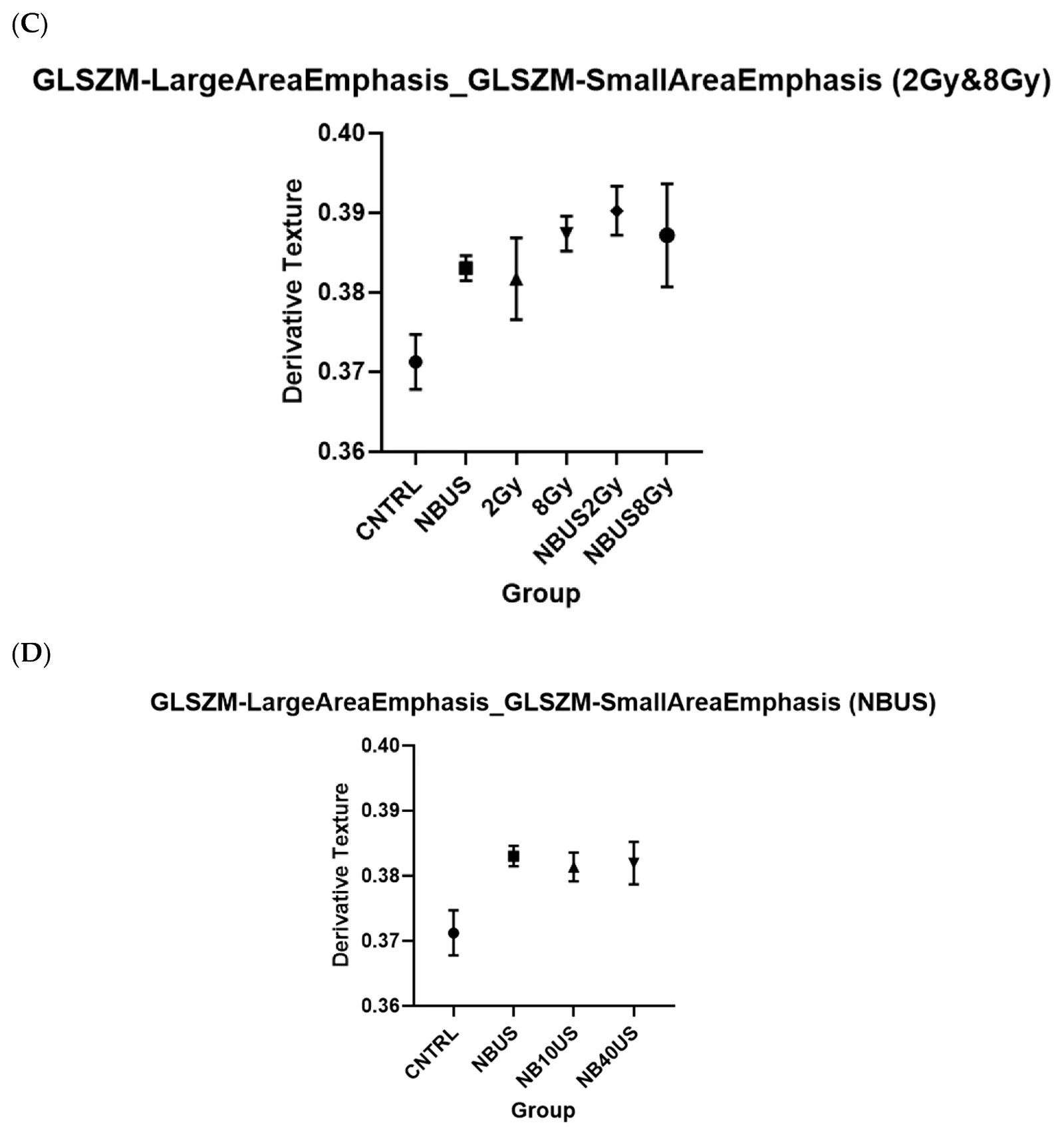
First-order texture analysis: (**A**) GLSZM_LargeAreaEmphasis (2Gy&8Gy); and (**B**) GLSZM_LargeAreaEmphasis (NBUS). Derivative texture analysis: (**C**) GLSZM_LargeAreaEmphasis—GLSZM_SmallAreaEmphasis (2Gy&8Gy); and (**D**) GLSZM_LargeAreaEmphasis—GLSZM_SmallAreaEmphasis (NBUS).

The time interval between NB injection and ultrasound activation (0 min, 10 min, and 40 min) was also investigated to evaluate the potential differences in vascular and peritumoral effects. As shown in Figure 5B, compared with the Control group, all NBUS treatment groups induced some degree of tumor cell death or apoptosis activation. However, NBUS appeared to induce apoptosis only to a limited extent, and variations in the injection-to-activation interval did not show significant differences.

The first-order texture feature GLSZM_LAE effectively indicated the treatment outcomes across different experimental conditions. However, in certain cases, GLSZM_LAE could not fully distinguish the differences between tissue states, such as regions with dense cell populations or necrotic zones, which may both lead to high GLSZM_LAE values due to their apparent local homogeneity. In order to address this limitation, a derivative texture analysis was employed. Based on the selection process, the derivative feature GLSZM_SAE on GLSZM_LAE was selected to distinguish between cases in which the cell status differs despite similar GLSZM_LAE values. As presented in Figure 5C, the derivative results for the various treatments followed a similar overall trend to that presented in Figure 5A. Specifically, NBUS only produced effects comparable to 2 Gy, while the combination treatment NBUS + 2Gy significantly enhanced the therapeutic outcome. In contrast, for the NBUS-only groups without irradiation, both the first-order and derivative texture analyses (as shown in Figure 5B,D) indicated no significant differences among the NBUS, NB10US, and NB40US conditions, suggesting that variations in the injection-to-activation interval exerted a minimal impact on the tumor response in the absence of radiation.

**Figure 6:**
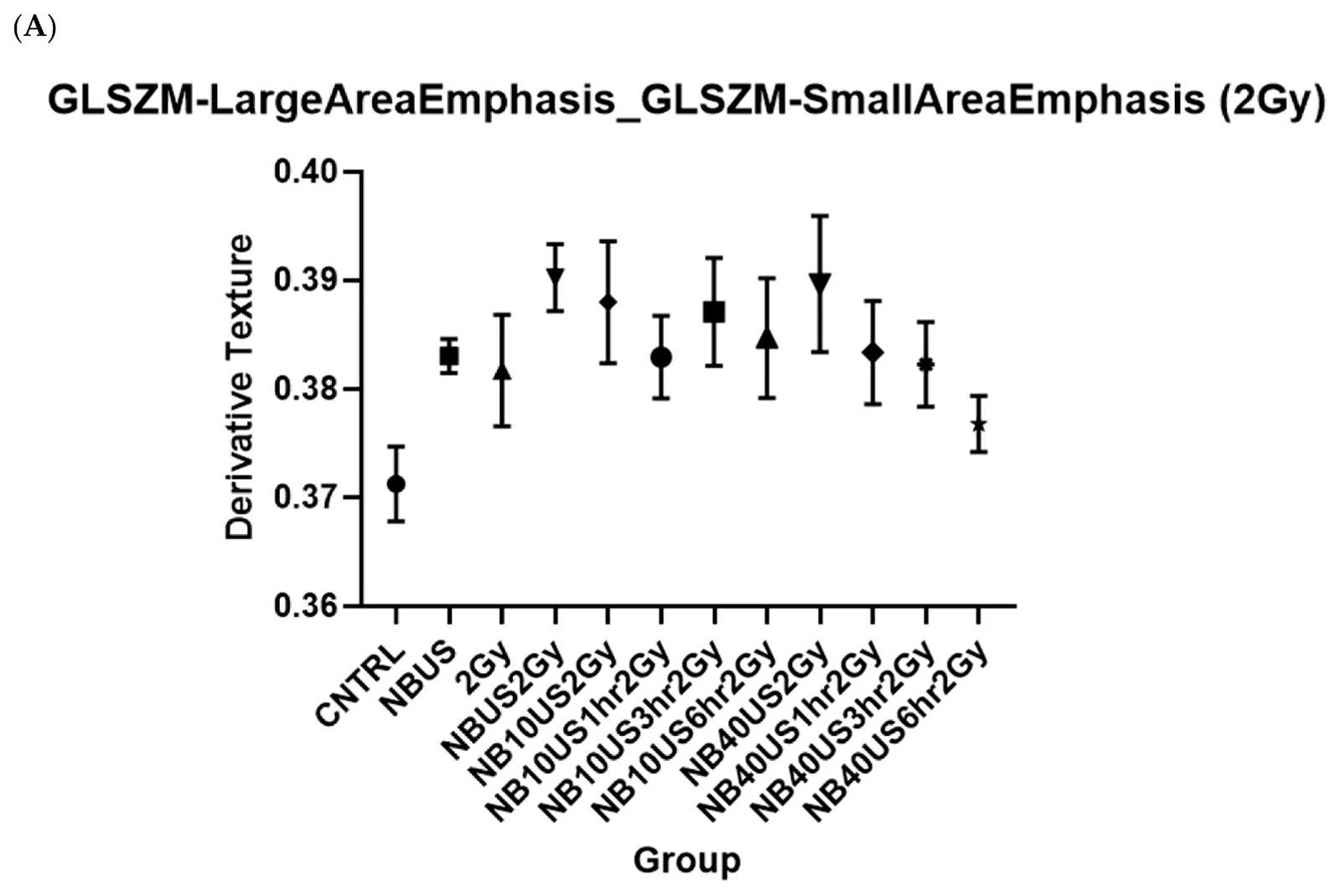

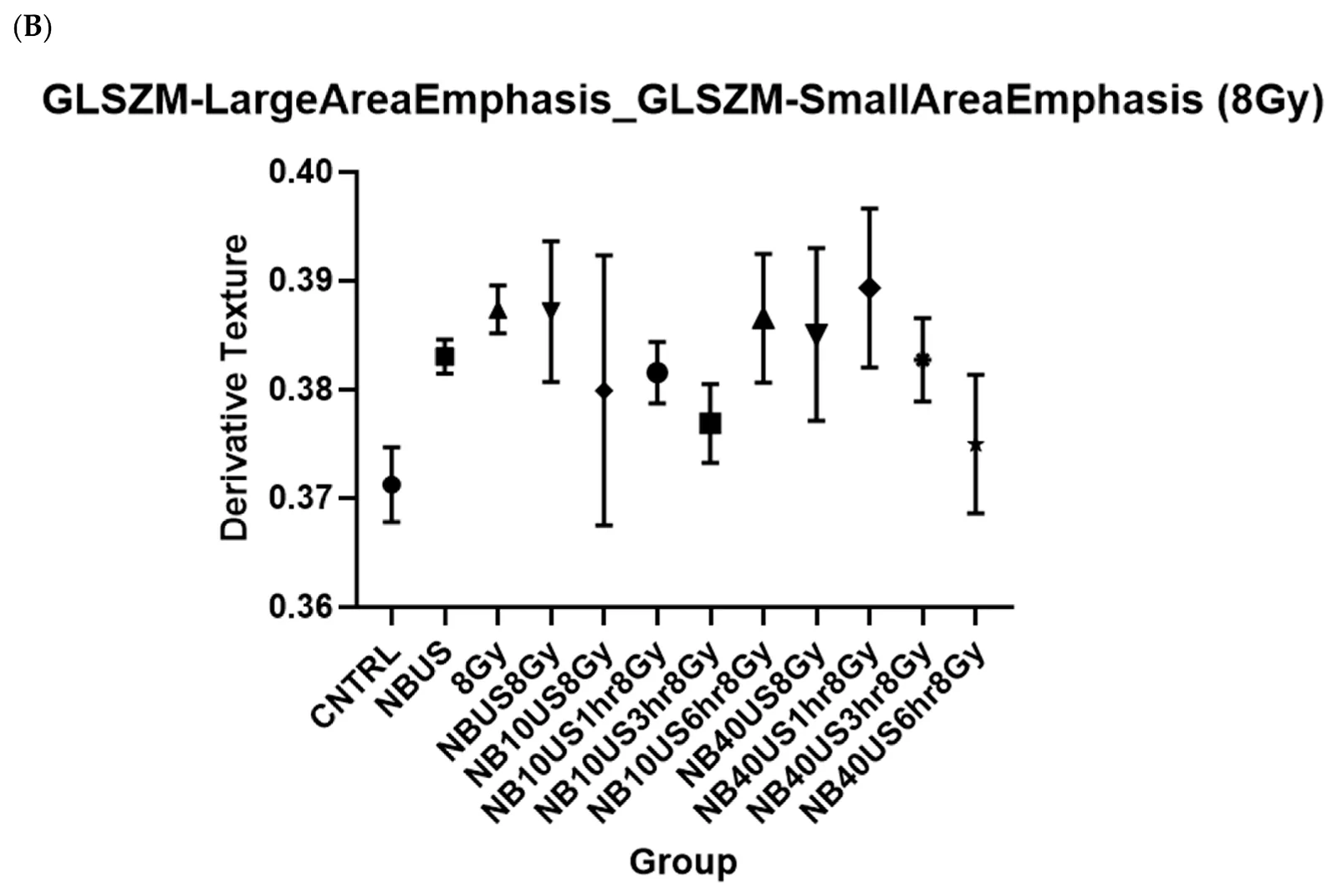
Optimization of time gap between treatments: (**A**) 2Gy; and (**B**) 8Gy.

The effects of time intervals between treatments at different radiation doses were evaluated, as shown in Figure 6. Two parameters were optimized: the interval between NB injection and ultrasound activation (0 min, 10 min, and 40 min), and the interval between NBUS treatment and subsequent radiation therapy (0 h, 1 h, 3 h, and 6 h). Both of them were tested for radiation doses of 2 Gy (Figure 6A) and 8 Gy (Figure 6B). An analysis based on the derivative texture feature GLSZM_SAE on GLSZM_LAE demonstrated that performing NBUS treatment prior to radiation effectively enhanced the tumor treatment effects to varying degrees. Specifically, at 2 Gy, radiation only produced a similar level of tumor damage as NBUS only, while, at 8 Gy, radiation only produced a higher level of tumor cell death than NBUS only, as expected. However, combining NBUS with 2 Gy resulted in significantly greater cellular disruption and apoptosis compared with either treatment individually, while the combined NBUS + 8 Gy treatment did not further increase cell death obviously. Studies conducted at 2 Gy demonstrated a higher stability and reliability than those at 8 Gy. Combination treatments involving 2 Gy were generally able to achieve better efficacy. To some extent, introducing a time interval between NB injection and US administration could influence the therapeutic effect. The results indicated that a 10-min delayed activation yielded effects comparable to immediate activation, while a 40-minute delay resulted in slightly improved therapeutic outcomes, which achieved a somewhat better efficacy. However, delayed radiotherapy following NB activation did not appear to yield any observable improvement in the outcomes. Combination treatments involving 8 Gy did not reach the desired effects. The introduction of NBUS prior to 8 Gy did yield slightly better therapeutic outcomes in some animals, but the considerable biological variation among individuals resulted in unstable treatment responses. As expected in animal studies, substantial biological variation among individuals were observed, likely due to genetic and environmental factors, which caused large error bars. Meanwhile, the limited sample size may also lead to an inflated standard deviation to some extent. Nevertheless, the consistent trends across groups remained statistically significant, suggesting that the different treatment modalities generated biologically relevant effects.

## 4. Discussion

The texture analysis of histology has emerged as a promising technique for evaluating tumor treatment outcomes. Derivative texture analysis provides a deeper approach to enhance both the sensitivity and accuracy in the analysis by looking for repeating patterns of textures. Here, in this study, derivative texture analysis was applied to digital images of H&E-stained slices. First-order texture analysis was performed directly on normalized gray-scaled and downsampled H&E images, whereas second-order texture analysis was applied to the texture maps generated in the previous step. A one-way ANOVA test and a k-NN classifier model were used to select the most informative features for distinguishing between the differences in treatment outcomes. Tukey’s multiple-comparisons testing was subsequently used to evaluate the significant differences between groups.

Based on feature selection using the one-way ANOVA test combined with a k-NN classifier, both feature 20 alone, and feature 20 in combination with features 1, 19, and 21 achieved predictive accuracies of tumor cell destruction exceeding 80%. However, a detailed comparison between H&E-stained tissue sections and the corresponding feature maps revealed that feature 21 had limited correspondence with the status of histological tissue. Moreover, the derivative features 1 and 19 exhibited substantial overlap between treatment groups. In contrast, feature 20 (GLSZM_SAE on GLSZM_LAE) demonstrated a strong consistency with the histopathological findings and provided a clear differentiation in treatment outcomes across groups. Therefore, feature 20 can be considered the most robust and biologically meaningful feature among the evaluated features in the work here.

GLSZM-based texture analysis is a method commonly used in quantitative image analysis, especially in medical imaging. It characterizes the spatial distribution of pixel intensities by considering both gray-level (intensity) values and the size of the connected zones of pixels with the same gray-level value. A zone in GLSZM is defined as a set of contiguous pixels sharing the same gray intensity. GLSZM captures both the intensity and the size of homogeneous regions, which can provide a promising way to quantify tissue heterogeneity. LAE measures the distribution of large homogeneous zones within an image. A high LAE value means the presence of large, uniform areas of similar intensity. In H&E-stained tumor slices, a high LAE may correspond to regions of dense healthy cell zones or necrotic zones, with large areas of cells or extracellular material present in uniform staining. SAE measures the distribution of small homogeneous zones within an image. There are high SAE value responses to fine texture or highly heterogeneous regions. In histology, an increase in the SAE value can indicate cellular heterogeneity, scattered apoptotic bodies, or fragmented tissue structure.

Radiation therapy can induce apoptosis, a key mechanism underlying its effectiveness in cancer treatment. It causes DNA damage, which activates the DNA damage response (DDR) pathway and triggers apoptosis to prevent the propagation of damaged cells. Then, it will lead to the typical morphological features of apoptosis: cell shrinkage, chromatin condensation, and the formation of apoptotic bodies. However, the therapeutic outcomes of radiation therapy are often limited by radioresistance, which remains one of the major challenges in radiotherapy. Several biological factors can cause radiation resistance, such as efficient DNA repair mechanisms, protective proteins, and high levels of antioxidants.

The main purpose of using MBs or NBs with ultrasound (US) prior to radiation therapy is to enhance tumor radiosensitivity. In the work here, NBs were applied to overcome radioresistance and increase the efficiency of radiation-induced cell death. The results indicated that NBUS treatment can significantly enhance the therapeutic outcomes of both 2 Gy and 8 Gy radiation doses compared to radiation only. The results demonstrated that tumors treated with NBUS + 2 Gy exhibited outcomes comparable to those treated with 8 Gy only, revealing that NBUS can effectively lower the radiation dose required to achieve similar therapeutic efficacy. This finding suggests a strong potential for using NBUS to achieve dose reduction, thereby minimizing the radiation-induced damage to the surrounding healthy tissues, one of the critical challenges in the clinical radiotherapy. Furthermore, although the NBUS + 8Gy group also presented a slight enhancement over 8 Gy only, the enhancement was less than that observed at the 2 Gy dose. This suggests that the radiosensitization effect of NBUS may have a limitation. Nevertheless, overall, the results indicate that there may be a limitation in the maximal enhancement achievable with radiation therapy and NBUS. Moreover, the more consistent outcomes observed at 2 Gy compared with 8 Gy further highlight the robustness and sensitivity of the GLSZM-derived metric in capturing the treatment-induced heterogeneity and optimizing combination therapy parameters.

In the analysis work here, healthy tumor cells without any treatment (control) are tightly packed and uniformly stained, which are dense and viable. This presents a large homogeneous area in H&E, which directly increases the LAE value. In contrast, tumor cells undergo apoptosis or even necrosis after different treatments, where the tumor tissue contains debris, karyorrhexis, vacuoles, hemorrhage, and irregular staining, which change the homogeneity, resulting in the LAE value decreasing [45]. However, the LAE does not measure the cells in health or death directly. It measures the homogeneity of large-scale staining. Not only can the viable dense tissue can result in high LAE values, but the dead tissue with necrosis can result in them as well [46,47]. Here, the derivative feature GLSZM_SAE on GLSZM_LAE was selected to address this limitation, where the second-order texture feature GLSZM_SAE is computed on the output of the texture feature GLSZM_LAE. In effect, applying the SAE after LAE acts as a higher-order filter, revealing microstructural disruptions that are covered in the first-order large-zone analysis. Compared with using the texture feature GLSZM_LAE alone, the strategy of using “texture-of-texture” makes the analysis more responsive to tissue changes. It enhances the detection of fine variations within otherwise large homogeneous areas, allowing the differentiation between bulk tissue (generally structurally organized and viable) and bulk necrotic/apoptotic areas (characterized by subtle fragmentation and internal heterogeneity). Therefore, the derivative feature 20 (GLSZM_SAE on GLSZM_LAE) can be identified as the most effective parameter for evaluating therapeutic outcomes across different treatment groups. An analysis based on this feature indicated that the combination therapy group presented a better treatment efficacy compared to both the 2 Gy only and NBUS only groups, as well as to the control group. Furthermore, activating NB via focused ultrasound at the tumor site produced therapeutic effects comparable to those achieved with 2 Gy only, indicating that NB, in combination with using US, has a great potential to induce apoptosis independently within the tumor region.

**Figure 7:**
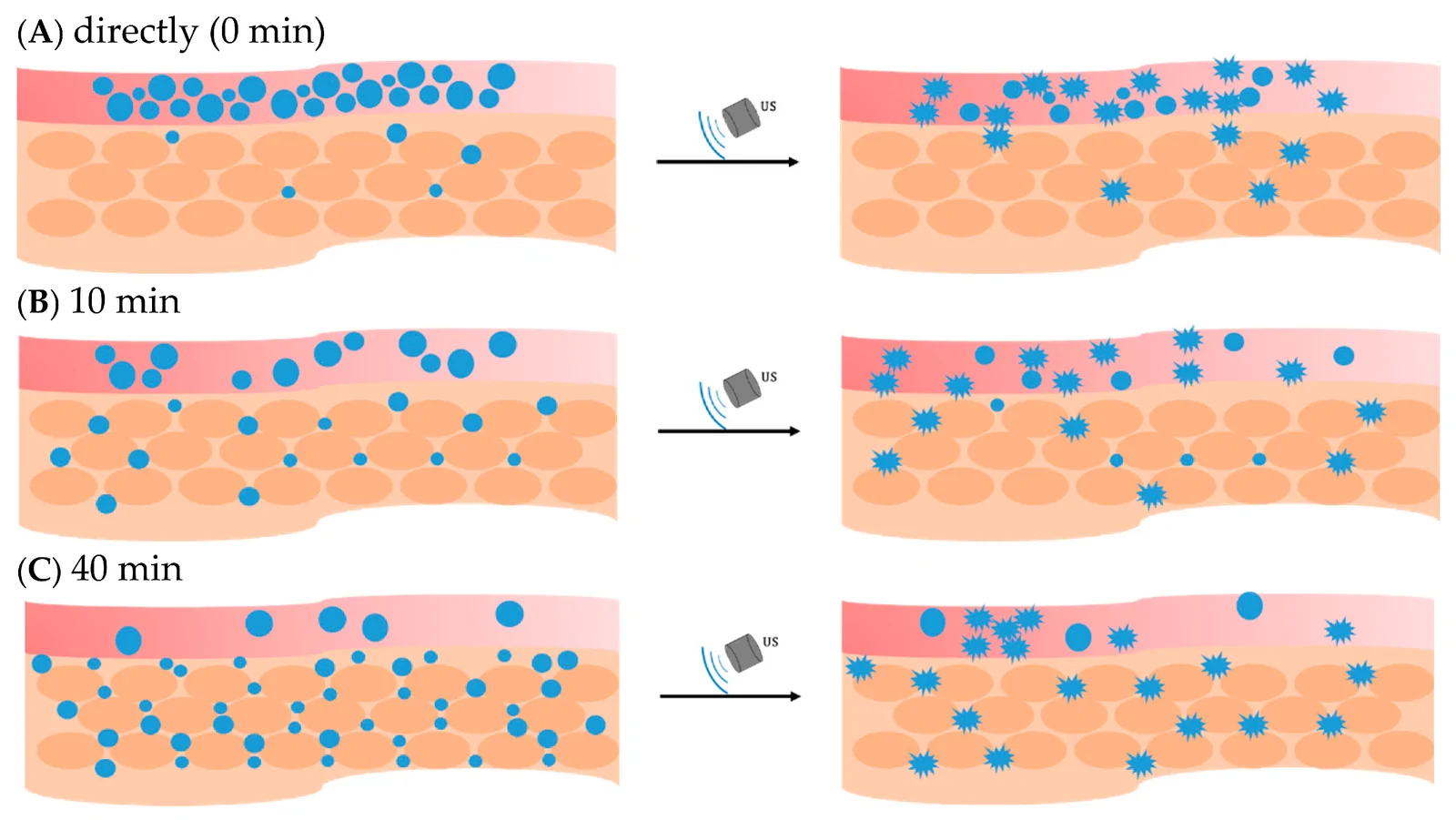
Scheme of NB activated by US after different period: (**A**) directly (0 min); (**B**) 10 min; and (**C**) 40 min.

Combining NBUS treatment with radiation therapy indicates that NBUS can enhance the effects of irradiation, leading to increased tumor cell death and greater vascular damage. Previous studies have demonstrated that NBs can passively accumulate within tumor tissue through the tumor vasculature and remain in the tumor for a longer period than MBs, resulting in an increased local retention over time [24,48]. Therefore, a longer interval between NB injection and US activation may allow more NB accumulation within the tumor, potentially enhancing the subsequent therapeutic response. As illustrated in Figure 7A, immediately after injection, US is applied while NBs are still circulating within the vasculature. Similar to MBs, these intravascular NBs enhance radiosensitivity primarily through vascular disruption and apoptosis induction. However, the smaller size of NBs can allow them to penetrate more deeply into the microvasculature. Histological texture analysis indicated that the NBUS-only treatment produced outcomes similar to 2 Gy only. As illustrated in Figure 7B, and described elsewhere, applying US 10 min after an NB injection provides sufficient time for smaller NBs to penetrate into the tumor interstitial space, while a large proportion of NBs of average or larger size remain within the blood vessels. This dual distribution leads to two damage mechanisms during US exposure: the intravascular NBs cause vascular disruption similar to MBs; the extravascular NBs directly interact with tumor cell membranes, and disturbing their integrity and enhancing radiation-induced apoptosis. As illustrated in Figure 7C, based on a histological texture analysis, applying US 40 min after NB injection appears to offer the optimal conditions for NBs to accumulate within the tumor region, allowing localized ultrasound activation to selectively enhance the radiosensitivity of cells within the targeted area, achieving a more focused therapeutic effect. As described elsewhere, applying US at different time points after NB injection, immediately (0 min), after 10 min, or after 40 min, corresponds to different biological damages. The immediate activation (0 min) primarily causes vascular damage only. The 10 min activation induces both vascular and tumor cell damage. The 40 min activation mainly results in direct tumor cell damage. The 0 min and 10 min activations mainly rely on vascular disruption, similar to the mechanism observed with MBUS, where endothelial cell death leads to impaired perfusion and delayed tumor growth. In contrast, activation at 40 min mainly targets tumor cells, directly triggering apoptotic pathways.

Our previous study investigated the changes in the cellular pattern in prostate cancer by texture analysis on hematoxylin and eosin (H&E)-stained histology images [43]. In that study, prostate cancer xenografts in a rabbit model were treated with radiation alone, MBUS alone, or a combination of both. The MBUS primarily causes vascular damage to enhance treatment efficacy. Texture analysis was applied on the segmented tumor cell damaged region. Among several texture analysis methods, the Gray-Level Size Zone Matrix (GLSZM) demonstrated the most significant treatment-induced changes in cellular patterns following USMB therapy. However, our current study is based on a mouse model with NB-assisted radiation therapy which can act more directly on tumor cells by directly triggering apoptotic pathways, and investigated effective texture features which can be used to evaluate NBUS treatment outcomes. Moreover, this PC3 xenograft model used in this study provides a promising platform for evaluating prostate cancer progression, tumor vascular responses, and the therapeutic effects of combining NBUS treatment with radiation therapy. We acknowledge that the differences in tumor biological response, vascular-mediated mechanisms, and immune status may affect treatment outcomes across cancer types. However, our current findings still provide important mechanistic insight into the potential of using NBUS to enhance XRT. Therefore, future studies using additional tumor models will be important to further the translational potential of using this therapeutic approach.

Emerging image-based AI models, including vision-enabled large language models (LLM), have been developed rapidly and demonstrated potential in tumor diagnosis and histopathological analysis. There is a great potential in incorporating this approach to future applications in the quantitative assessment of treatment histological changes, such as apoptosis, necrosis, vascular disruption, and tissue heterogeneity, which are difficult to evaluate consistently via a visual inspection. These models may further contribute to the individualized optimization of the treatment response and support individualized therapy timing and dosing strategies in future clinical practices.

## 5. Conclusions

This study demonstrates that texture analysis is a reliable technique for differentiating treatment outcomes across different therapeutic approaches. Among the evaluated features, GLSZM_SAE on GLSZM_LAE was selected as the most effective feature for characterizing the treatment response. Using a k-NN classifier model, the prediction achieved accuracy, sensitivity, and precision exceeding 80%, with a specificity reaching 93.65%. The findings were consistent with work indicating that activating NBs using focused ultrasound at the tumor region before receiving radiation therapy can enhance the therapeutic efficacy by leading to increased apoptosis and better treatment outcomes compared to radiation only. This provides potential for reducing radiation exposure to obtain the treatment purpose while effectively protecting healthy tissues during treatment. The findings support work that shows that directly targeting tumor cell membranes may be a more effective strategy than inducing endothelial damage alone. The textural analysis of standard haematoxylin-and-eosin-stained histology samples can be used to analyze the complex biology involved in the responses.

## Figures and Tables

**Table 1:** Overview of the four computational methods.

Method Name	Measures	Focuses	Captures
**GLCM**	Frequency of gray-level pairs at a certain distance and direction.	Spatial relationship between two pixels.	Local patterns and symmetry.
**GLDM**	Number of neighboring pixels that are similar to the center pixel.	Pixel dependence and neighborhood similarity.	Texture granularity/smoothness.
**GLSZM**	Size of connected areas (zones) with the same gray level.	Homogeneous region size.	Uniform region distribution.
**NGTDM**	Difference between a pixel and the average gray level of its neighborhood.	Global contrast and texture smoothness/roughness.	Global structure and contrast.

**Table 2:** Overview of experimental groups.

No.	Treatment	Number of Mice
**1**	Control	5
**2**	2 Gy	5
**3**	8 Gy	4
**4**	Nanobubble + Ultrasound (NBUS)	5
**5**	Nanobubble + Ultrasound + 2 Gy (NBUS2Gy)	6
**6**	Nanobubble + Ultrasound+8 Gy (NBUS8Gy)	3
**7**	Nanobubble + 10 min + Ultrasound (NB10US)	4
**8**	Nanobubble + 10 min + Ultrasound + 2 Gy (NB10US2Gy)	4
**9**	Nanobubble + 10 min + Ultrasound + 1 h + 2 Gy (NB10US1hr2Gy)	4
**10**	Nanobubble + 10 min + Ultrasound + 3 h + 2 Gy (NB10US3hr2Gy)	4
**11**	Nanobubble + 10 min + Ultrasound + 6 h + 2 Gy (NB10US6hr2Gy)	4
**12**	Nanobubble + 10 min + Ultrasound + 8 Gy (NB10US8Gy)	3
**13**	Nanobubble + 10 min + Ultrasound + 1 h + 8 Gy (NB10US1hr8Gy)	3
**14**	Nanobubble + 10 min + Ultrasound + 3 h + 8 Gy (NB10US3hr8Gy)	3
**15**	Nanobubble + 10 min + Ultrasound + 6 h + 8 Gy (NB10US6hr8Gy)	3
**16**	Nanobubble + 40 min + Ultrasound (NB40US)	5
**17**	Nanobubble + 40 min + Ultrasound + 2 Gy (NB40US2Gy)	5
**18**	Nanobubble + 40 min + Ultrasound + 1 h + 2 Gy (NB40US1hr2Gy)	3
**19**	Nanobubble + 40 min + Ultrasound + 3 h + 2 Gy (NB40US3hr2Gy)	4
**20**	Nanobubble + 40 min + Ultrasound + 6 h + 2 Gy (NB40US6hr2Gy)	3
**21**	Nanobubble + 40 min + Ultrasound + 8 Gy (NB40US8Gy)	3
**22**	Nanobubble + 40 min + Ultrasound + 1 h + 8 Gy (NB40US1hr8Gy)	3
**23**	Nanobubble + 40 min + Ultrasound + 3 h + 8 Gy (NB40US3hr8Gy)	3
**24**	Nanobubble + 40 min + Ultrasound + 6 h + 8 Gy (NB40US6hr8Gy)	3
**In total**		**92**

**Table 3:** Table of feature selection (one-way ANOVA).

No.	First-Order Feature	Second-Order Feature	*p* Value
**1**	original_glcm_InverseVariance	original_glcm_Correlation	0.009
**2**	original_glcm_InverseVariance	original_glcm_Imc1	0.009
**3**	original_glcm_JointAverage	original_glcm_Contrast	0.041
**4**	original_glcm_JointAverage	original_glcm_DifferenceVariance	0.035
**5**	original_gldm_DependenceEntropy	original_gldm_DependenceVariance	0.002
**6**	original_gldm_GrayLevelNonUniformity	original_gldm_DependenceVariance	0.019
**7**	original_gldm_LargeDependenceEmphasis	original_gldm_DependenceVariance	0.048
**8**	original_gldm_LargeDependenceLowGrayLevelEmphasis	original_gldm_DependenceEntropy	0.012
**9**	original_gldm_LargeDependenceLowGrayLevelEmphasis	original_gldm_GrayLevelNonUniformity	0.045
**10**	original_gldm_LargeDependenceLowGrayLevelEmphasis	original_gldm_GrayLevelVariance	0.022
**11**	original_gldm_LargeDependenceLowGrayLevelEmphasis	original_gldm_HighGrayLevelEmphasis	0.006
**12**	original_gldm_LargeDependenceLowGrayLevelEmphasis	original_gldm_LargeDependenceHighGrayLevelEmphasis	0.030
**13**	original_gldm_LargeDependenceLowGrayLevelEmphasis	original_gldm_SmallDependenceHighGrayLevelEmphasis	0.048
**14**	original_gldm_LowGrayLevelEmphasis	original_gldm_DependenceEntropy	0.042
**15**	original_gldm_LowGrayLevelEmphasis	original_gldm_GrayLevelNonUniformity	0.030
**16**	original_gldm_SmallDependenceEmphasis	original_gldm_DependenceVariance	0.003
**17**	original_glszm_GrayLevelNonUniformity	original_glszm_LargeAreaLowGrayLevelEmphasis	0.022
**18**	original_glszm_GrayLevelNonUniformityNormalized	original_glszm_SizeZoneNonUniformityNormalized	0.039
**19**	original_glszm_LargeAreaEmphasis	original_glszm_SizeZoneNonUniformityNormalized	0.00002
**20**	original_glszm_LargeAreaEmphasis	original_glszm_SmallAreaEmphasis	0.000001
**21**	original_glszm_LargeAreaHighGrayLevelEmphasis	original_glszm_SizeZoneNonUniformityNormalized	0.007
**22**	original_glszm_LargeAreaHighGrayLevelEmphasis	original_glszm_SmallAreaEmphasis	0.006
**23**	original_glszm_LargeAreaLowGrayLevelEmphasis	original_glszm_GrayLevelNonUniformityNormalized	0.022
**24**	original_glszm_LargeAreaLowGrayLevelEmphasis	original_glszm_HighGrayLevelZoneEmphasis	0.018
**25**	original_glszm_LargeAreaLowGrayLevelEmphasis	original_glszm_LargeAreaEmphasis	0.038
**26**	original_glszm_LargeAreaLowGrayLevelEmphasis	original_glszm_SmallAreaHighGrayLevelEmphasis	0.010
**27**	original_glszm_LargeAreaLowGrayLevelEmphasis	original_glszm_ZoneVariance	0.039
**28**	original_glszm_LowGrayLevelZoneEmphasis	original_glszm_GrayLevelNonUniformityNormalized	0.030
**29**	original_glszm_SmallAreaHighGrayLevelEmphasis	original_glszm_SizeZoneNonUniformityNormalized	0.045
**30**	original_glszm_SmallAreaHighGrayLevelEmphasis	original_glszm_SmallAreaEmphasis	0.041
**31**	original_glszm_ZoneEntropy	original_glszm_SizeZoneNonUniformityNormalized	0.003
**32**	original_glszm_ZoneEntropy	original_glszm_SmallAreaEmphasis	0.003

**Table 4:** Feature selection results for k-NN classifier: (**A**) K = 1; (**B**) K = 2; (**C**) K = 3; and (**D**) K = 4.

(**A**)				(**B**)		
**K = 1**				**K = 2**		
**Number of** **Features Used**	**Number of** **Features Used**	**Number of** **Features Used**		**Number of** **Features Used**	**Features** **Combination**	**%Accuracy**
1	20	80.95		1	20	80.95
2	18, 20	66.67		2	1, 20	61.90
3	18, 19, 20	71.43		3	1, 20, 31	61.90
4	18, 19, 20, 31	57.14		4	1, 19, 20, 21	80.95
5	1, 18, 19, 20, 31	52.38		5	1, 19, 20, 21, 23	71.43
6	1, 18, 19, 20, 21, 31	66.67		6	1, 18, 19, 20, 21, 31	66.67
7	1, 18, 19, 20, 22, 23, 29	57.14		7	1, 18, 19, 20, 23, 28, 32	47.62
8	1, 18, 19, 20, 22, 23, 29, 31	42.86		8	1, 19, 20, 21, 22, 23, 30, 31	33.33
(**C**)				(**D**)		
**K = 3**				**K = 4**		
**Number of** **Features Used**	**Number of** **Features Used**	**Number of** **Features Used**		**Number of** **Features Used**	**Features** **Combination**	**%Accuracy**
1	20	80.95		1	30	33.33
2	18, 20	66.67		2	1, 20	61.90
3	18, 19, 20	71.43		3	20, 21, 31	71.43
4	1, 18, 19, 20	66.67		4	1, 19, 30, 32	71.43
5	1, 18, 19, 20, 30	42.86		5	1, 18, 19, 20, 30	42.86
6	1, 18, 19, 20, 29, 30	47.62		6	1, 18, 19, 20, 29, 30	47.62
7	1, 19, 20, 21, 22, 23, 31	52.38		7	1, 18, 19, 20, 29, 30, 31	47.62
8	18, 19, 20, 21, 22, 23, 31, 32	42.86		8	1, 19, 20, 21, 29, 30, 31, 32	38.10

**Table 5:** Results of selected feature (*k*-NN): (**A**) feature 20; and (**B**) combination of feature 1, 19, 20, and 21.

(**A**)				
**Group**	**%Sn**	**%Sp**	**%Precision**	**%Accuracy**
**Control**	80.00	100.00	100.00	-
**NBUS**	100.00	93.75	83.33	-
**2 Gy**	60.00	87.50	60.00	-
**NBUS2Gy**	83.33	93.33	83.33	-
**Macro Average**	80.83	93.65	81.67	-
**Micro Average**	80.95	93.65	80.95	-
**Overall**	-	-	-	**80.95**
(**B**)				
**Group**	**%Sn**	**%Sp**	**%Precision**	**%Accuracy**
**Control**	80.00	100.00	100.00	-
**NBUS**	40.00	92.86	66.67	-
**2 Gy**	83.33	96.43	83.33	-
**NBUS2Gy**	100.00	89.29	75.00	-
**Macro Average**	75.83	94.64	81.25	-
**Micro Average**	80.95	94.64	80.95	-
**Overall**	-	-	-	**80.95**

**Table 6:** Tukey’s multiple-comparisons test of select feature.

	Feature 1	Feature 19	Feature 20	Feature 21
Tukey’s Multiple Comparisons Test	*p* Value	*p* Value	*p* Value	*p* Value
**CNTRL vs. NBUS**	0.8770	0.0028	0.0003	0.4742
**CNTRL vs. 2Gy**	0.3986	0.0031	0.0011	0.0060
**CNTRL vs. NBUS2Gy**	0.0080	<0.0001	<0.0001	0.8064
**NBUS vs. 2Gy**	0.8224	>0.9999	0.9306	0.1144
**NBUS vs. NBUS2Gy**	0.0396	0.0697	0.0170	0.9171
**2Gy vs. NBUS2Gy**	0.2069	0.0638	0.0046	0.0271

## Data Availability

The raw data for this project can be made available by Gregory J. Czarnota upon request.
